# Characterization of chiral amino acids from different milk origins using ultra-performance liquid chromatography coupled to ion-mobility mass spectrometry

**DOI:** 10.1038/srep46289

**Published:** 2017-04-10

**Authors:** He Tian, Nan Zheng, Songli Li, Yangdong Zhang, Shengguo Zhao, Fang Wen, Jiaqi Wang

**Affiliations:** 1 State Key Laboratory of Animal Nutrition, Ministry of Agriculture Laboratory of Quality& Safety Risk Assessment for Dairy Products (Beijing), Institute of Animal Science, Chinese Academy of Agricultural Sciences, Beijing, 100193, P.R. China

## Abstract

Milk contains free amino acids (AAs) that play essential roles in maintaining the growth and health of infants, and D-AA isomers are increasingly being recognized as important signalling molecules. However, there are no studies of the different characteristics of chiral AA (C-AA) from different milk origins. Here, UPLC coupled to ion-mobility high-resolution MS (IM-HRMS) was employed to characterize 18 pairs of C-AAs in human, cow, yak, buffalo, goat, and camel milk. The results proved that milk origins can be differentiated based on the D- to L- AA ratio-based projection scores by principal component analysis. The present study gives a deeper understanding of the D- to L- AA ratio underlying the biological functions of different animal milks, and provide a new strategy for the study of AA metabolic pathways.

Free amino acids (AAs), which consist of mirror-image configuration L- and D- AA isomers, are essential biological molecules with fundamental roles in maintaining the growth and health of the body. Moreover, D-AAs are increasingly being recognized as important signaling molecules in diseases^1^,^2^,^3^ and as new biologically active substances^4^,^5^. Some D-AAs are synthesized via currently unidentified metabolic routes. Alterations in D-AA concentrations have been implicated in the pathogenesis of several human diseases, including amyotrophic lateral sclerosis^6^,^7^. The role of D-serine as an endogenous ligand for the N-methyl-D-aspartate receptor in the central nervous system has been elucidated. D-aspartate has been proposed to play a role in the survival and development of newborn hippocampal neurons, learning and memory, hormone regulation, and spermatogenesis^6^,^8^,^9^,^10^,^11^.

Milk contains chiral AAs (C-AAs) that are important to ensure optimal growth, neurodevelopment, and health^12^,^13^. People consume D-AAs daily, and some of the nutritional effects have been identified^14^. However, no studies have been performed to characterize the different compositions of L- and D- AAs and their underlying biological functions in human, cow, yak, buffalo, goat, and camel milk to enable the selection of the appropriate milk source based on an individual body’s needs.

Previously reported detection methods, such as capillary electrophoresis, micellar electrokinetic chromatography, an enzyme based biosensor, and derivatization HPLC, lack specificity and sensitivity compared with LC/GC-MS^15^,^16^,^17^,^18^,^19^,^20^,^21^. In terms of sensitivity and dynamic range for quantification, triple quadrupole (QqQ) tandem MS is universally recognized as superior to high-resolution (HR) MS^22^. However, QqQ tandem MS, even in MRM mode, is not sufficiently selective and can be affected by complex matrices and background noises due to its unit mass resolution^22^,^23^. The recently increased sophistication of HRMS instruments has greatly enhanced the ion transmission efficiency, scan speed, detector sensitivity, dynamic range, and linearity^22^, and it has already exhibited advantages over QqQ MS with respect to multi-target analysis and avoidance of false positives from complex matrices^23^,^24^,^25^,^26^. Nevertheless, HRMS is incapable of identifying target compounds from co-eluting isomers or matrix with identical *m/z* values.

A promising strategy to overcome these limitations is ultra-performance liquid chromatography (UPLC) coupled to the combination of ion mobility (IM) and MS^27^. In IM, ions migrate through a cell, filled with an inert neutral buffer gas in electric fields^28^, and the drift time required to pass through this electric field depend on the ion charge state, size, and structure of the analyte. In addition to chromatographic separation, and HR precursor and product ion *m/z* data, IM offers a third analytical dimension for selecting target ions based on their drift time, thus greatly improving the selectivity and signal-to-noise ratio for target compound identification. IM–HRMS has proven to be valuable in the structural and quantitative analysis of both small and large molecules^29^,^30^,^31^,^32^,^33^,^34^,^35^,^36^,^37^.

In the present study, a method of UPLC coupled to IM–HRMS for quantifying C-AAs in milk was developed and applied to the analysis of the characteristics of L- and D-AAs in human, cow, yak, buffalo, goat, and camel milk. An overview of the characteristics of the study is illustrated in Fig. 1.

## Results and Discussion

The LC-MS parameters for the 19 pairs of derivatized C-AAs and their stable-isotope labelled standards are listed in Table 1, including theoretical *m/z*, retention time (RT), measured *m/z*, and accuracy. It has been reported that (S)-NIFE derivatization can achieve D- and L-AA separations^20^, which has also been realized in the present study for all of 19 pairs of C-AAs (Table 1).

**Figure 2:**
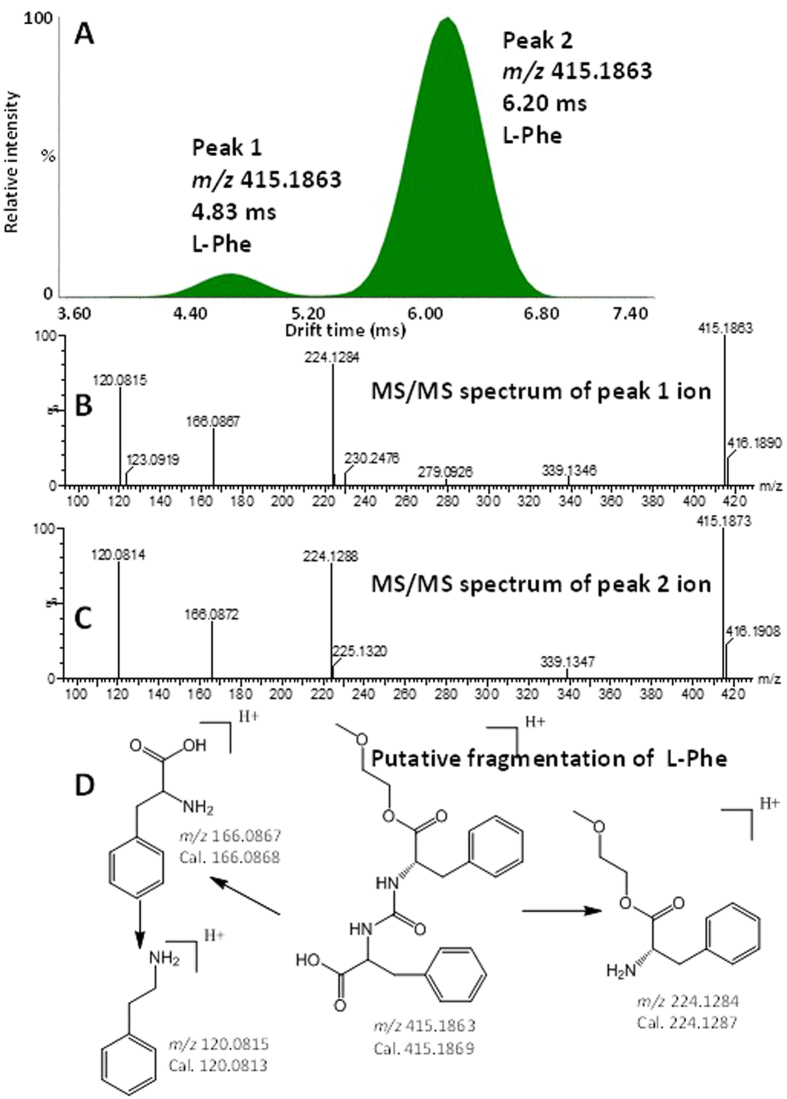
Two peaks from single L-Phe in IM and their MS/MS spectra and putative fragmentation. (**A**) MS/MS spectra of the peak 1 ion. (**B**) MS/MS spectra of the peak 2 ion. (**C**) MS/MS spectra of the peak 2 ions, respectively. (**D**) Putative fragmentation of derivatized L-Phe.

The selectivity of the method was assessed using 10 underivatized milk samples from each milk origin. The lack of a signal greater than 3 times the signal-to-noise ratio at the same elution time as the target compound indicated the absence of a false positive signal from matrix interference. An interesting discovery was found that the detection of derivatized L-phenylalanine (L-Phe) using UPLC-IMMS displayed peaks in different drift time (dt), see Fig. 2A. It has been reported that small ions may have multiple conformers in the gas phase to produce multiple peaks in IM^38^. The production of multiple peaks is thought to be the result of protonation site isomers. Further MS/MS analysis of these two ions showed that their product ion spectra was almost the same (Fig. 2B,C), and Fig. 2D is the putative fragmentations of derivatized L-Phe. Thereby, it is proved that these two peaks came from a single analyte.

It has been reported that based on a doping drift gas with a volatile chiral reagent, IMMS can separate multiple pairs of C-AAs, including D- and L-tryptophan, D- and L-methionine, D- and L-threonine, D- and L-phenylalanine, and D- and L-serine^27^. However, these five L-AAs were not sufficiently separated from their respective D-enantiomers for accurate quantification, even after the drift gas of an IMMS was modified with a chiral vapor^27^. Therefore, the present study used the (S)-NIFE derivatization method to separate D- and L-AAs during gradient chromatographic elutions, and peak overlapping was not observed for any enantiomeric pairs. Then, MS data acquisition for every AA was divided into different scan functions with window widths of ±0.3 min around the retention time. Window widths of any scan functions for L-AAs were never overlapped with their respective D-enantiomers. In this way, accurate quantitation can also be achieved, even for a pair of C-AAs that have the same dt.

Eight-point standard calibration curves of spiked milk (0.04–1000 ng/mL) were used for the evaluation of the calibration curve linearity. The precision of this method was assessed using 6 replicate analyses at 3 spiked levels on 9 consecutive days. The results listed in Table S1 indicated good linearity for all C-AA quantifications with R^2^ values greater than 0.99, and good precision with %RSDs less than 6.4%.

The present method trueness was evaluated using recovery analyses of each analyte in milk at three levels ranging from 0.4 ng/mL to 1000 ng/mL, with 6 replicates on 9 consecutive days. Overall, the extraction recoveries for all spiked analytes in milk were in the range of 82.1–105.2% (Table S1).

The matrix effects for all the C-AAs were within ±20% (Table S1), indicating acceptable^39^.

LODs were calculated in quintuplicate using blank samples spiked from 0.01 to 20 ng/mL, depending on the detection sensitivity for each analyte, and the LODs ranged from 0.01 ng/mL for methionine to 6.14 ng/mL for serine (Table S1).

In comparison to Q-TOF, the Q-IM-TOF mode improved signal-to-noise ratio more than 2-fold, demonstrating the advantages of the third analytical dimension in excluding matrix or background interference (Fig. S1).

The present method was applied to quantify C-AAs in milk samples from human, cow, yak, buffalo, goat, and camel (Table S2). Although L- and D- AAs are mirror-image configuration isomers, they play different roles and have various D- to L- AA ratios (DLAArs) that are dependent on their biological utilization and function as well as the body’s requirements. The DLAArs of all 18 pairs of C-AAs were calculated (Table 2).

**Figure 3:**
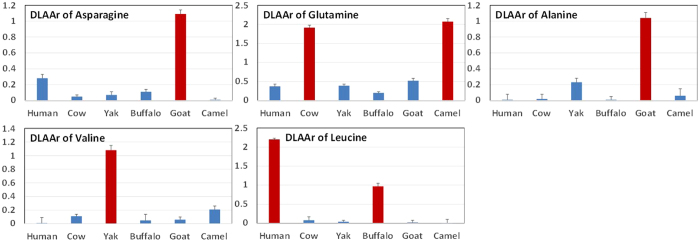
Characteristic D- to L- AA ratio (DLAArs) in milk from human, cow, yak, buffalo, goat, and camel. The DLAArs in milk origins that reached or exceeded 1.0 were asparagine in goat, glutamine in cow and camel, alanine in goat, valine in yak, and leucine in human and buffalo.

**Figure 4:**
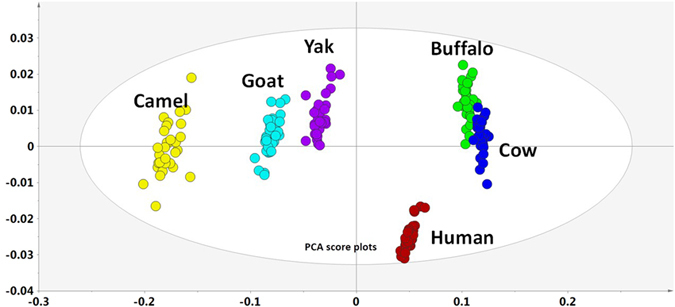
Principal component analysis was conducted to study the characteristics of DLAArs based on milk origin. Milk origins were differentiated by the D- to L- AA ratio.

The DLAArs were constant within individual milk origin with relative standard deviations less than 0.10 (Table 2). Most D-AA concentrations were substantially lower than L-AA concentrations. However, an interesting discovery was that the DLAArs of some AAs of certain milk origins were distinct, reaching or exceeding 1.0, including asparagine in goat, glutamine in cow and camel, alanine in goat, valine in yak, and leucine in human and buffalo (Fig. 3). Principal component analysis was conducted to study the characteristics of the DLAArs based on the milk origin, and clustering and grouping were observed in the score plots (Fig. 4). This clustering represents constant physiological intra-group states, and the grouping in the score plots indicated that the bioavailability and biological utilization of C-AAs vary widely between different milk origins. The enzymes, involved in D-AA synthesis and metabolism include D-AA oxidases, deacylases, dehydrogenases, epimerases, proteases, and racemases, the activities of which may determine the DLAArs of the different milk origins^2^,^37^,^38^,^39^,^40^,^41^,^42^,^43^,^44^.

Some D-AAs have important biological functions. D-asparagine aggravates nephritis in rats induced by Staphylococcus aureus bacteria^45^, and prevents K and Mg depletion in rats induced by diuretics^46^. Glutamine, the principal carrier of nitrogen in the body, is synthesized from glutamate and ammonia and is an important energy source for many cells. Changes in the D-alanine content of the rat pancreas are related to their diurnal and nocturnal (circadian) habits^47^. Administration of total parenteral nutrition containing D-leunine, D-methionine, D-phenylalanine, and D-valine to hepatoma-bearing rats showed that D-valine inhibits tumor growth without negative effects to the host. D-leunine also improved the nutritional status of the sick rats^48^. These observations suggest that some diets rich in D-AA may benefit cancer patients. Various DLAArs of the different milk origins depend on the biological utilization and needs of the body.

Concentration changes of D-AAs in milk can be linked to pathophysiological conditions of the body. It has been reported that the ratio of free D-AAs to free AAs increased significantly in milk of cows with mastitis^49^. Although the somatic cell count (SCC) in milk is the routine index of mastitis in dairy stock, the measure of D-AAs in milk can facilitate a deeper understanding of mastitis pathogenesis and its effects on lactation. As different feedstuff exert influences on AA compositions in milk^50^, specific analysis of L-AAs and D-AAs may provide deep insight into metabolic mechanism of dairy animals and humans. The significant discrepancy of the concentrations of some D-AAs in cow milk between the present research and a published study^18^ may be a reflection of differences of microorganism numbers between both samples^51^.

Metabolomics have been widely applied to the discovery of disease biomarkers and pathophysiological mechanisms, and some AAs are regarded as potential biomarkers for early diagnosis of cancers and other diseases^52^,^53^. However, these studies did not indicate whether L- or D- AAs were perturbed, which would provide additional biological information underlying pathophysiological alterations. Thus, it is strongly recommended that future studies of biomarkers and metabolic pathways of diseases related to AAs should obtain specific results regarding L- and D- AAs.

## Conclusion

Compared with LC-Q-TOF instruments, the three analytical dimensions of LC-Q-IM-TOF combined with the retention time, *m/z*, and drift time can better and specifically identify target compounds while excluding the interference of co-eluting isomers or matrix ions with identical *m/z*, and enhance the signal-to-noise ratio to improve the sensitivity of the C-AAs’ quantification. The present study of C-AAs in human, cow, yak, buffalo, goat, and camel milk showed that milk DLAArs can be used to detect the differences in milk origin and to indicate different biological utilization and function of C-AAs from different milk origins, and DLAArs may reflect the pathophysiological status of body.

## Materials and Methods

All experiments involving animals were conducted according to the principles of the Chinese Academy of Agricultural Sciences Animal Care and Use Committee (Beijing, China), which approved the study protocols. All human participants provided informed consent through signed forms, and the methods were performed in accordance with the relevant guidelines.

### Chemicals and reagents

The stable-isotope labelled compounds were purchased from Cambridge Isotope Laboratories (Andover, MA, USA). Standards of L- and D- amino acids were obtained from Sigma–Aldrich (St. Louis, MO, USA). All standards, chemicals, reagents, and solvents used in this study are fully described in the Supporting Information.

### Sample collection

Human milk samples from healthy volunteers were obtained following informed consent and approval of the local ethics committee. Thirty fasting milk samples were respectively collected from human, cow, yak, buffalo, goat, and camel at the mid-lactating stage, and the indexes of temperature and humidity were approximately 50–60. All samples were stored at −80 °C until analyses.

### Sample preparation

A 500 μL sample of milk was mixed with 100 μL of internal standard (IS) solution, which contained 80 μM of each of the stable-isotope-labelled AAs. After 10 min of incubation on ice, 2000 μL of ice-cold acetonitrile was added, the solution was vortexed, and the mixture was incubated for at least 15 min on ice. Any precipitate formed was removed by centrifugation (5 min, 20,000 × g). The supernatant was directly loaded onto an SPE cartridge (Oasis PRiME HLB 60 mg, 3 mL; Waters, Milford, MA, USA). This SPE has been newly developed by the Waters Company to remove lipids from biological samples, and does not need the traditional processes of preequilibration, binding, and washing, requiring only one step of filtration. All elutes were collected into a centrifuge tube and were evaporated to dryness under gentle ultra-high-purity nitrogen gas at 40 °C. Based on a published paper^20^, a derivatization reagent of (S)-NIFE was introduced into present study to separate D- and L-AA during the chromatographic elution. The residue was redissolved with 100 μL of water, followed by the addition of 70 μL of 0.15 M sodium tetraborate and 100 μL of a 2.5 mg/mL (S)-NIFE solution in acetonitrile. This mixture was incubated at room temperature for 10 min. The reaction was terminated by the addition of 20 μL of 4 M hydrogen chloride and 710 μL of water. The reactions solutions were then filtered (Econofltr PES, 0.2 μm), and diluted with water. A set of standard solutions containing L- and D- AAs and the IS solution was processed in parallel. Calibration standards were prepared by spiking derivative standard solutions and IS solutions into underivatized milk.

### Method validation

The validation protocol was based on the commission decision 2002/657/EEC (Commission Decision 2002/657/EC, 2002) to evaluate the identification, confirmation, matrix-effect, dynamic range, linearity, precision, limits of detection (LODs), and limits of quantification (LOQs). The LODs were set to 3.3 times the signal-to-noise ratio in the blank matrix, and 10 times the signal-to-noise ratio was used as the LOQs.

The target compounds were identified and confirmed using the retention time window, which was obtained as the mean retention time ± three standard deviations of the retention time of ten blank samples spiked at concentrations of 5 times the LOQs (Fig. S1)^54^. The high-resolution precursor ion *m/z*, isotope abundance ratio, MS/55, and drift time were also used for the identification.

The matrix effects (ME%) were studied by comparing the slope of the matrix-matched calibration curves with the slope of the matrix-free calibration curves. Samples were first extracted and prepared according to the procedure described above. The matrix effect was investigated by calculating the percentage (ME%) of signal enhancement or suppression according to equation ME% = (S_s_/S_m_ − 1) × 100, where S_s_ is the slope of the calibration plot of the matrix-matched calibration solutions and S_m_ is the slope of the calibration plot of the solvent standards.

### UPLC-IM-HRMS analysis

The procedure used a Waters Acquity UPLC system coupled to a Synapt G2-Si HDMS travelling-wave quadrupole/ion mobility/orthogonal acceleration time-of-flight mass spectrometer (Waters MS Technologies, Manchester, UK). Five microliters of the prepared sample was injected into a column at 60 °C (ACQUITY UPLC BEH C_18_ 1.7 μm, 2.1 × 100 mm column, Waters, Dublin, Ireland). The temperature of the auto-sampler was 10 °C. The gradient consisted of mobile phase A (10 mM ammonium hydrogencarbonate in water, pH = 9.5) and mobile phase B (ACN) pumped at 0.6 mL/min with a total run time of 24 min. The linear gradients were: 96% A at 5 min, 90% A at 9 min, 88.5% A at 11 min, 72% A at 16 min, 70% A at 19 min, 65% A at 21 min, 0% A at 21.1 min, 0% A at 23 min, 96% A at 23.1 min, and 96% A at 24 min.

The mass spectrometer was operated in positive electrospray ionization mode. The sample cone voltage, extraction cone voltage, source temperature, desolvation temperature, desolvation gas flow and cone gas flow were optimized. Leucine enkephalin was used as the lock mass [M + H]^+^ at *m/z* 556.2771. A sodium formate solution was used for external instrument calibration. The scan range was from 50 to 1000 *m/z*. The start and end time for data acquisition were divided into different functions with window widths of ±0.3 min around the retention time of the target compounds. In this way, we were able to extract the IM peak areas of the AAs without interference from their individual chiral isomers or background ions with an identical *m/z* and drift time. The product ion scan was used for confirmation of the target compounds.

The parameters of the IM experiments of the trap gas flow, helium cell gas flow, IMMS gas flow, wave height, trap DC bias, and IM wave velocity were 2 mL/min, 180 mL/min, 90 mL/min, 40 V, 45 V and 1000 m/s, respectively. The pressures inside the helium and the IMMS cells under the experimental conditions were 5.00 and 2.85 mbar, respectively. The IMMS data analysis was performed using MassLynx 4.1, DriftScope 2.4 (Waters Corporation).

## Additional Information

**How to cite this article:** Tian, H. *et al*. Characterization of chiral amino acids from different milk origins using ultra-performance liquid chromatography coupled to ion-mobility mass spectrometry. *Sci. Rep.* **7**, 46289; doi: 10.1038/srep46289 (2017).

**Publisher's note:** Springer Nature remains neutral with regard to jurisdictional claims in published maps and institutional affiliations.

## Supplementary Material

Supplementary Information

## Figures and Tables

**Figure 1:**
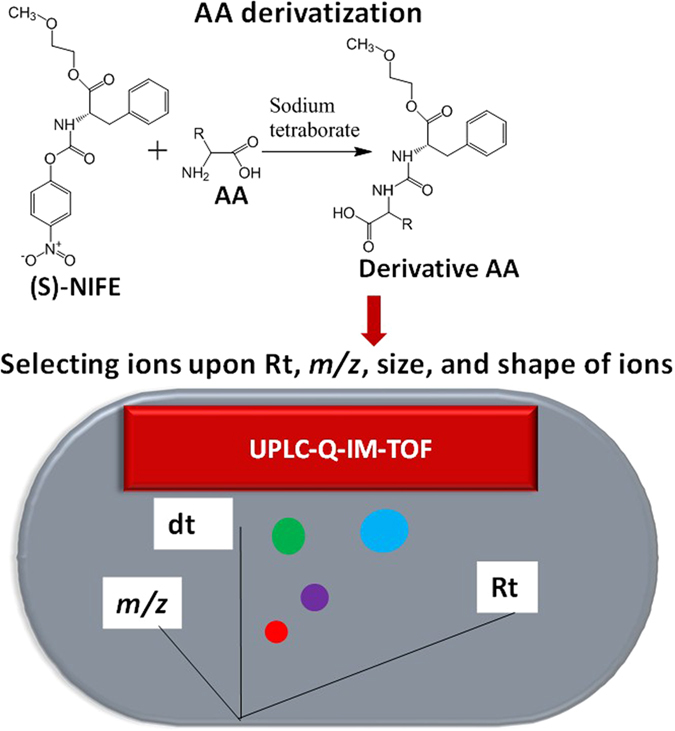
Overview of the characteristics of the presents study on investigating the compositions of C-AAs in human, cow, yak, buffalo, goat, and camel milk. IMMS, ion mobility and mass spectrometry; Rt, retention time; dt, drift time; C-AA, chiral amino acid; PCA, principle component analysis.

**Table 1:** LC**-**MS parameters for analyzing chiral amino acids. RT, retention time.

Compounds	Derivatized Element Composition	Theoretical (*m*/*z*)	L-Enantiomer			D-Enantiomer		
			RT (min)	Measured (*m*/*z*)	Accuracy (ppm)	RT (min)	Measured (*m*/*z*)	Accuracy (ppm)
Glutamate	C_18_H_24_N_2_O_8_	397.1605	6.01	397.1605	0.00	6.51	397.1610	1.26
D_5_-Glutamate	D_5_C_18_H_19_N_2_O_8_	402.1919	6.01	402.1925	1.49			
Aspartate	C_17_H_22_N_2_O_8_	383.1449	6.20	383.1451	0.52	6.50	383.1455	1.57
D_3_-Aspartate	D_3_C_17_H_19_N_2_O_8_	386.1637	6.19	386.1646	2.33			
Asparagine	C_17_H_23_N_3_O_7_	382.1609	9.41	382.1613	1.05	9.94	382.1611	0.52
^13^C_4_-Asparagine	^13^C_4_C_13_H_23_N_3_O_7_	386.1743	9.41	386.1745	0.52			
Serine	C_16_H_22_N_2_O_7_	355.1500	9.77	355.1501	0.28	10.25	355.1500	0.00
D_3_-Serine	D_3_C_16_H_19_N_2_O_7_	358.1688	9.76	358.1684	−1.12		358.1685	−0.83
Glutamine	C_18_H_25_N_3_O_7_	396.1765	9.88	396.1766	0.25	10.44	396.1764	−0.25
D_5_-Glutamine	D_5_C_18_H_20_N_3_O_7_	401.2079	9.86	401.2077	−0.50			
Histidine	C_19_H_24_N_4_O_6_	405.1769	10.17	405.1770	0.25	10.83	405.1772	0.74
D_5_-Histidine	D_5_C_19_H_19_N_4_O_6_	410.2082	10.15	410.2081	−0.24			
Threonine	C_17_H_24_N_2_O_7_	369.1656	10.29	369.1653	−0.81	11.85	369.1655	−0.27
D_5_-Threonine	D_5_C_17_H_19_N_2_O_7_	374.1970	10.30	374.1965	−1.34			
Alanine	C_16_H_22_N_2_O_6_	339.1551	10.50	339.1554	0.89	11.97	339.1553	0.59
D_7_-Alanine	D_7_C_16_H_15_N_2_O_6_	346.1996	10.50	346.1995	−0.29			
Arginine	C_19_H_29_N_5_O_6_	424.2191	10.81	424.2198	1.65	11.46	424.2197	1.41
D_7_-Arginine	D_7_C_19_H_22_N_5_O_6_	431.2630	10.76	431.2634	0.93			
Proline	C_18_H_24_N_2_O_6_	365.1707	11.93	365.1709	0.54	12.50	365.1704	−0.82
D_7_-Proline	D_7_C_18_H_17_N_2_O_6_	372.2147	11.84	372.2146	−0.27			
Valine	C_18_H_26_N_2_O_6_	367.1864	12.98	367.1866	0.55	14.21	367.1867	0.82
D_8_-Valine	D_8_C_18_H_18_N_2_O_6_	375.2366	12.94	375.2363	−0.80	14.17	375.2359	
Methionine	C_18_H_26_N_2_O_6_S	399.1584	13.54	399.1587	0.75	14.45	399.1582	−0.50
D_3_-Methionine	D_3_C_18_H_23_N_2_O_6_S	402.1773	13.50	402.1780	1.74			
Isoleucine	C_19_H_28_N_2_O_6_	381.2020	14.13	381.2018	−0.53	15.08	381.2023	0.79
D_10_-Isoleucine	D_10_C_19_H_18_N_2_O_6_	391.2648	14.09	391.2646	−0.51			
Leucine	C_19_H_28_N_2_O_6_	381.2020	14.40	381.2021	0.26	15.35	381.2022	0.52
D_10_-Leucine	D_10_C_19_H_18_N_2_O_6_	391.2648	14.35	391.2646	−0.51			
Phenylalanine	C_22_H_26_N_2_O_6_	415.1863	15.04	415.1859	−0.96	15.85	415.1868	1.20
D_8_-Phenylalanine	D_8_C_22_H_18_N_2_O_6_	423.2366	15.00	423.2368	−0.47			
Tryptophan	C_24_H_27_N_3_O_6_	454.1973	15.26	454.1970	−0.66	15.94	454.1969	−0.88
D_8_-Tryptophan	D_8_C_24_H_19_N_3_O_6_	462.2475	15.22	462.2480	1.08			
Cystine	C_32_H_42_N_4_O_12_S_2_	739.2313	15.83	739.2321	1.08	16.82	739.2318	0.68
D_2_-Cystine	D_2_C_32_H_40_N_4_O_12_S_2_	741.2439	15.84	741.2433	−0.81			
Lysine	C_32_H_44_N_4_O_10_	645.3130	17.45	645.3127	−0.47	17.92	645.3129	−0.16
D_9_-Lysine	D_9_C_32_H_35_N_4_O_10_	654.3695	17.42	654.3694	−0.15			
Tyrosine	C_35_H_41_N_3_O_11_	680.2814	20.09	680.2816	0.29	20.82	680.2813	−0.15
D_7_-Tyrosine	D_7_C_35_H_34_N_3_O_11_	687.3259	20.10	687.3269	1.46			

**Table 2:** Ratio of D-AA to L- AA from different milk origins.

Compounds	Human	Cow	Yak	Buffalo	Goat	Camel
Aspartate	0.29 ± 0.07	0.23 ± 0.07	0.35 ± 0.10	0.20 ± 0.05	0.19 ± 0.05	0.19 ± 0.07
Asparagine	0.28 ± 0.06	0.05 ± 0.00	0.07 ± 0.02	0.10 ± 0.03	1.09 ± 0.06	0.01 ± 0.00
Serine	0.01 ± 0.00	0.07 ± 0.00	0.20 ± 0.07	0.34 ± 0.04	0.02 ± 0.00	0.01 ± 0.00
Glutamine	0.37 ± 0.06	1.90 ± 0.07	0.39 ± 0.04	0.20 ± 0.03	0.52 ± 0.06	2.07 ± 0.08
Histidine	0.08 ± 0.02	0.30 ± 0.05	0.22 ± 0.04	0.55 ± 0.08	0.22 ± 0.03	0.30 ± 0.05
Threonine	0.01 ± 0.00	0.07 ± 0.02	0.31 ± 0.07	0.88 ± 0.08	0.33 ± 0.04	0.01 ± 0.00
Alanine	0.01 ± 0.00	0.02 ± 0.00	0.23 ± 0.05	0.01 ± 0.00	1.04 ± 0.07	0.06 ± 0.01
Arginine	0.05 ± 0.01	0.02 ± 0.00	0.06 ± 0.01	0.73 ± 0.08	0.22 ± 0.03	0.05 ± 0.00
Proline	0.02 ± 0.00	0.03 ± 0.00	0.23 ± 0.03	0.10 ± 0.02	0.05 ± 0.03	0.08 ± 0.02
Valine	0.01 ± 0.00	0.11 ± 0.03	1.08 ± 0.07	0.05 ± 0.00	0.06 ± 0.02	0.21 ± 0.05
Methionine	0.12 ± 0.03	0.24 ± 0.06	0.08 ± 0.02	0.13 ± 0.04	0.02 ± 0.00	0.17 ± 0.06
Isoleucine	0.06 ± 0.00	0.07 ± 0.02	0.16 ± 0.04	0.03 ± 0.00	0.33 ± 0.05	0.13 ± 0.02
Leucine	2.20 ± 0.09	0.08 ± 0.03	0.03 ± 0.00	0.97 ± 0.08	0.02 ± 0.00	0.01 ± 0.00
Phenylalanine	0.05 ± 0.00	0.09 ± 0.02	0.06 ± 0.00	0.06 ± 0.01	0.15 ± 0.03	0.12 ± 0.03
Tryptophan	0.06 ± 0.00	0.02 ± 0.00	0.04 ± 0.00	0.05 ± 0.00	0.03 ± 0.00	0.04 ± 0.00
Cystine	0.28 ± 0.04	0.37 ± 0.07	0.49 ± 0.05	0.08 ± 0.02	0.10 ± 0.02	0.19 ± 0.04
Lysine	0.01 ± 0.00	0.04 ± 0.00	0.01 ± 0.00	0.05 ± 0.00	0.02 ± 0.00	0.01 ± 0.00
Tyrosine	0.02 ± 0.00	0.31 ± 0.08	0.02 ± 0.00	0.10 ± 0.03	0.21 ± 0.04	0.32 ± 0.07
