# Supplementary material for: Characterization of chiral amino acids from different milk origins using ultra-performance liquid chromatography coupled to ion-mobility mass spectrometry
Source: Sci Rep. 2017 Apr 10;7:46289. doi: 10.1038/srep46289 (PMC5385494; doi:10.1038/srep46289)
Supplement: Supplementary Information [file srep46289-s1.doc]

**Characterization of chiral amino acids from different milk origins using ultra-performance liquid chromatography coupled to ion-mobility mass spectrometry**

He Tian**^*^**, Nan Zheng**^*^**, Songli Li, Yangdong Zhang, Shengguo Zhao, Fang Wen, and Jiaqi Wang

State Key Laboratory of Animal Nutrition, Ministry of Agriculture Laboratory of Quality& Safety Risk Assessment for Dairy Products (Beijing), Institute of Animal Science, Chinese Academy of Agricultural Sciences, Beijing, 100193, P.R. China. ^*^These authors contributed equally to this work. Correspondence and requests for materials should be addressed to J.W. (email: [wang-jia-qi@263.net](mailto:wang-jia-qi@263.net)).

**Supplementary Information**

Chemicals and Reagents.

**Tables**

**1.** Table S1 - Limits of detection (LOD), accuracy, intra-assay precision and inter-assay precision (RSD%) for L- and D-amino acid measurements in human, cow, yak, buffalo, goat, and camel milk

**2.** Table S2 - Concentrations of C-AA (μg/mL) in different milk origins.

**Figure S1** - Comparison of the detection sensitivity of amino acids between Q-TOF and Q-IM-TOF.

**Chemicals and Reagents**

The stable-isotope labeled compounds were purchased from Cambridge Isotope Laboratories (Andover, MA, USA), including [2,3,3,4,4-D_5_]-L-glutamate, [2,3,3-D_3_]-L-aspartate, [^13^C_4_]-L-asparagine, [2,3,3-D_3_]-L-serine, [2,3,3,4,4-D_5_]-L-glutamine, [Ring-2,4-D_2_; Alpha, Beta, Beta-D_3_]-L-histidine, [D_5_]-L-threonine, [D_7_]-L-alanine, [D_7_]-L-arginine, [D_7_]-L-proline, [D_8_]-L-valine, [D_3_]-L-methionine, [D_10_]-L-isoleucine, [D_10_]-L-leucine, [D_8_]-L-phenylalanine, [D_8_]-L-tryptophan, [3,3-D_2_]-L-cystine, [D_9_]-L-lysine, [D_7_]-L-tyrosine. L- and D- amino acids were obtained from Sigma–Aldrich (St. Louis, MO, USA), containing L-glutamate, D-glutamate, L-aspartate, D-aspartate, L-asparagine, D- asparagine, L-serine, D-serine, L-glutamine, D-glutamine, L-histidine, D-histidine, L-threonine, D-threonine, L-alanine, D-alanine, L-arginine, D-arginine, L-proline, D-proline, L-valine, D-valine, L-methionine, D-methionine, L-isoleucine, D-isoleucine, L-leucine, D-leucine, L-phenylalanine, D-phenylalanine, L-tryptophan, D-tryptophan, L-cystine, D-cystine, L-lysine, D-lysine, L-tyrosine, D-tyrosine. Ammonium bicarbonate and ammonium hydroxide solution of LC-MS grade, sodium tetraborate (99.998%), and hydrogen chloride (ACS reagent) were also obtained from Sigma–Aldrich (St. Louis, MO, USA). N-(4-nitrophenoxycarbonyl)-l-phenylalanine 2-methoxyethyl ester ((S)-NIFE) was purchased from Santa Cruz Biotechnology, Inc. (United States). Acetonitrile (ACN) and methanol (MeOH) of LC-MS grade, were from Fisher Chemicals (Fair Lawn, NJ).

**Table S1**. Limits of detection (LOD), accuracy, intra-assay precision and inter-assay precision (RSD%) for L- and D-amino acid measurements in milk.

| Compounds | | LOD/LOQ  (ng mL^-1^) | Dynamic range  (ng mL^-1^) | Linearity (*r*)^2^ | Matrix Effect (%) | RSD% at three levels  Spiked Recovery (%) | | (n = 9 days)  RSD% |
| --- | --- | --- | --- | --- | --- | --- | --- | --- |
| Glutamate |  | 0.06/0.18 | 0.20-50.00 | 0.9974 | -15 | 0.20 | 87.3 | 3.6 |
|  |  |  |  |  |  | 2.00 | 89.5 | 2.3 |
|  |  |  |  |  |  | 20.00 | 92.5 | 3.0 |
| Aspartate |  | 6.17/18.52 | 20.00-500.00 | 0.9926 | 8 | 20.00 | 91.7 | 4.9 |
|  |  |  |  |  |  | 100.00 | 92.9 | 3.9 |
|  |  |  |  |  |  | 500.00 | 93.4 | 3.3 |
| Asparagine |  | 0.52/1.57 | 2.00-150.00 | 0.9967 | 3 | 1.50 | 82.1 | 6.2 |
|  |  |  |  |  |  | 15.00 | 81.0 | 2.2 |
|  |  |  |  |  |  | 150.00 | 84.9 | 2.5 |
| Serine |  | 5.71/17.12 | 20.00-1000.00 | 0.9994 | 12 | 20.00 | 86.4 | 4.9 |
|  |  |  |  |  |  | 100.00 | 83.6 | 4.0 |
|  |  |  |  |  |  | 500.00 | 90.9 | 3.6 |
| Glutamine |  | 0.66/1.98 | 2.00-500.00 | 0.9920 | -6 | 2.00 | 86.1 | 3.5 |
|  |  |  |  |  |  | 20.00 | 83.7 | 2.2 |
|  |  |  |  |  |  | 200.00 | 89.4 | 0.7 |
| Histidine |  | 0.03/0.09 | 0.10-50.00 | 0.9955 | 10 | 0.10 | 92.6 | 2.7 |
|  |  |  |  |  |  | 2.00 | 91.0 | 4.0 |
|  |  |  |  |  |  | 40.00 | 95.7 | 3.9 |
| Threonine |  | 4.16/12.48 | 15.00-500.00 | 0.9982 | 9 | 15.00 | 86.2 | 3.6 |
|  |  |  |  |  |  | 60.00 | 88.3 | 4.2 |
|  |  |  |  |  |  | 240.00 | 87.5 | 3.6 |
| Alanine |  | 1.29/3.87 | 5.00-500.00 | 0.9979 | 17 | 5.00 | 100.7 | 6.4 |
|  |  |  |  |  |  | 50.00 | 103.5 | 5.1 |
|  |  |  |  |  |  | 500.00 | 106.8 | 3.8 |
| Arginine |  | 0.06/0.18 | 0.20-100.00 | 0.9999 | -13 | 0.20 | 93.6 | 6.0 |
|  |  |  |  |  |  | 4.00 | 90.7 | 0.9 |
|  |  |  |  |  |  | 80.00 | 94.9 | 1.8 |
| Proline |  | 0.06/0.18 | 0.20-100.00 | 0.9926 | -6 | 0.20 | 85.4 | 3.1 |
|  |  |  |  |  |  | 4.00 | 88.6 | 4.5 |
|  |  |  |  |  |  | 80.00 | 89.3 | 5.2 |
| Valine |  | 0.91/2.73 | 3.00-300.00 | 0.9921 | 6 | 3.00 | 94.8 | 5.4 |
|  |  |  |  |  |  | 30.00 | 103.5 | 2.1 |
|  |  |  |  |  |  | 300.00 | 101.7 | 1.3 |

**Table S1**. continued

| Compounds | | LOD/LOQ  (ng mL^-1^) | Dynamic range  (ng mL^-1^) | Linearity (*r*)^2^ | Effect (%) | RSD% at three levels  Spiked Recovery (%) | | (n = 9 days)  RSD% |
| --- | --- | --- | --- | --- | --- | --- | --- | --- |
| Methionine |  | 0.01/0.03 | 0.03-10.00 | 0.9900 | 2 | 0.03 | 83.4 | 4.7 |
|  |  |  |  |  |  | 0.3 | 85.9 | 3.8 |
|  |  |  |  |  |  | 3 | 87.7 | 3.9 |
| Isoleucine |  | 0.05/0.15 | 0.20-500.00 | 0.9979 | 14 | 0.15 | 82.1 | 1.8 |
|  |  |  |  |  |  | 3.00 | 84.5 | 3.2 |
|  |  |  |  |  |  | 60.00 | 86.7 | 2.6 |
| Leucine |  | 0.04/0.12 | 0.15-500.00 | 0.9947 | 19 | 0.15 | 90.3 | 3.0 |
|  |  |  |  |  |  | 3.00 | 92.4 | 2.8 |
|  |  |  |  |  |  | 60.00 | 95.6 | 3.9 |
| Phenylalanine |  | 0.16/0.48 | 0.50-200.00 | 0.9987 | 18 | 0.50 | 89.7 | 5.6 |
|  |  |  |  |  |  | 10.00 | 86.2 | 5.2 |
|  |  |  |  |  |  | 200.00 | 88.5 | 2.9 |
| Tryptophan |  | 0.04/0.12 | 0.15-500.00 | 0.9901 | -4 | 0.15 | 95.7 | 3.3 |
|  |  |  |  |  |  | 3.00 | 100.6 | 4.8 |
|  |  |  |  |  |  | 60.00 | 98.9 | 2.7 |
| Cystine |  | 5.89/17.67 | 20.00-1000.00 | 0.9997 | -10 | 20.00 | 103.4 | 1.9 |
|  |  |  |  |  |  | 100.00 | 101.5 | 3.4 |
|  |  |  |  |  |  | 500.00 | 100.3 | 5.1 |
| Lysine |  | 0.05/0.15 | 0.20-500.00 | 0.9946 | 12 | 0.15 | 89.4 | 5.9 |
|  |  |  |  |  |  | 3.00 | 92.1 | 3.7 |
|  |  |  |  |  |  | 60.00 | 93.0 | 2.8 |
| Tyrosine |  | 2.98/8.94 | 10.00-1000.00 | 0.9994 | 7 | 10.00 | 99.8 | 4.4 |
|  |  |  |  |  |  | 100.00 | 104.3 | 5.1 |
|  |  |  |  |  |  | 1000.00 | 105.2 | 3.2 |

**Table S2**. Concentrations of CAA (μg/mL) in different milk origins

| Compounds | Human | Cow | Yak | Buffalo | Goat | Camel |
| --- | --- | --- | --- | --- | --- | --- |
| L-Aspartate | 1.52 ± 0.15 | 0.97 ± 0.39 | 0.54 ± 0.13 | 0.69 ± 0.26 | 0.88 ± 0.17 | 0.37 ± 0.11 |
| D-Aspartate | 1.78 ± 0.13 | 0.22 ± 0.08 | 0.19 ± 0.04 | 0.14 ± 0.03 | 0.17 ± 0.06 | 0.07 ± 0.02 |
| L-Asparagine | 0.32 ± 0.09 | 0.22 ± 0.14 | 0.28 ± 0.25 | 0.86 ± 0.17 | 1.93 ± 0.21 | 2.28 ± 0.19 |
| D-Asparagine | 0.09 ± 0.03 | 0.01 ± 0.00 | 0.02 ± 0.01 | 0.09 ± 0.02 | 2.11 ± 0.34 | 0.01 ± 0.00 |
| L-Serine | 9.32 ± 0.07 | 0.27 ± 0.06 | 0.35 ± 0.04 | 6.47 ± 0.53 | 7.01 ± 0.75 | 7.34 ± 1.14 |
| D-Serine | 0.13 ± 0.04 | 0.02 ± 0.01 | 0.07 ± 0.02 | 2.23 ± 0.92 | 0.17 ± 0.08 | 0.02 ± 0.01 |
| L-Glutamine | 25.79 ± 3.41 | 51.96 ± 7.51 | 12.31 ± 2.77 | 20.93 ± 5.68 | 14.39 ± 5.48 | 33.25 ± 6.32 |
| D-Glutamine | 9.60 ± 5.26 | 98.97 ± 10.17 | 4.74 ± 1.94 | 4.26 ± 1.19 | 7.42 ± 2.39 | 68.88 ± 10.41 |
| L-Histidine | 22.20 ± 4.98 | 18.97 ± 8.29 | 13.62 ± 3.69 | 15.26 ± 4.33 | 10.59 ± 3.62 | 16.32 ± 4.65 |
| D-Histidine | 1.87 ± 0.35 | 5.74 ± 1.10 | 2.99 ± 0.88 | 8.34 ± 2.13 | 2.33 ± 0.98 | 4.89 ± 0.89 |
| L-Threonine | 3.34 ± 0.94 | 0.58 ± 0.13 | 0.16 ± 0.03 | 0.08 ± 0.02 | 2.09 ± 0.42 | 1.45 ± 0.33 |
| D-Threonine | 0.03 ± 0.01 | 0.04 ± 0.01 | 0.05 ± 0.01 | 0.07 ± 0.03 | 0.68 ± 0.14 | 0.02 ± 0.00 |
| L-Alanine | 8.62 ± 0.82 | 2.14 ± 0.21 | 0.93 ± 0.32 | 6.79 ± 0.59 | 6.51 ± 0.73 | 3.11 ± 0.08 |
| D-Alanine | 0.06 ± 0.01 | 0.05 ± 0.01 | 0.21 ± 0.03 | 0.08 ± 0.02 | 6.75 ± 0.48 | 0.19 ± 0.05 |
| L-Arginine | 1.11 ± 0.03 | 0.47 ± 0.06 | 0.35 ± 0.02 | 1.12 ± 0.21 | 1.13 ± 0.04 | 0.94 ± 0.09 |
| D-Arginine | 0.05 ± 0.01 | 0.01 ± 0.00 | 0.02 ± 0.01 | 0.82 ± 0.12 | 0.25 ± 0.04 | 0.05 ± 0.01 |
| L-Proline | 39.45 ± 5.77 | 73.65 ± 15.16 | 49.88 ± 77.42 | 60.71 ± 10.91 | 32.46 ± 67.23 | 12.14 ± 2.84 |
| D-Proline | 0.87 ± 0.14 | 1.95 ± 0.26 | 11.55 ± 2.53 | 6.34 ±1.65 | 1.47 ± 0.28 | 1.03 ± 0.17 |
| L-Valine | 89.91 ± 13.15 | 46.78 ± 13.24 | 66.74 ± 21.54 | 57.35 ± 8.34 | 71.22 ± 15.48 | 39.67 ± 6.21 |
| D-Valine | 1.24 ± 0.27 | 5.34 ± 1.59 | 71.95 ± 10.03 | 2.93 ± 1.15 | 4.36 ± 1.26 | 8.22 ± 3.40 |
| L-Methionine | 0.74 ± 0.14 | 0.17 ± 0.07 | 0.79 ± 0.13 | 0.23 ± 0.02 | 2.03 ± 0.13 | 0.12 ± 0.02 |
| D-Methionine | 0.09 ± 0.16 | 0.04 ± 0.01 | 0.06 ± 0.01 | 0.03 ± 0.01 | 0.04 ± 0.02 | 0.02 ± 0.00 |
| L-Isoleucine | 29.28 ± 5.39 | 13.14 ± 5.48 | 19.67 ± 8.54 | 11.15 ± 3.77 | 20.34 ± 5.42 | 32.22 ± 5.48 |
| D-Isoleucine | 1.87 ± 0.17 | 0..87 ± 0.13 | 3.13 ± 0.36 | 0.34 ± 0.08 | 6.79 ± 0.94 | 4.16 ± 0.91 |
| L-Leucine | 21.32 ± 2.75 | 10.67 ± 3.20 | 13.68 ± 1.76 | 23.44 ± 3.75 | 29.17 ± 4.13 | 15.15 ± 3.14 |
| D-Leucine | 46.93 ± 8.38 | 0.88 ± 0.12 | 0.42 ± 0.03 | 22.73 ± 4.26 | 0.70 ± 0.18 | 0.16 ± 0.06 |
| L-Phenylalanine | 356.82 ± 28.72 | 287.21 ± 44.31 | 403.27 ± 67.98 | 243.01 ± 86.47 | 299.14 ± 48.76 | 321.46 ± 95.77 |
| D-Phenylalanine | 16.58 ± 5.29 | 26.79 ± 6.85 | 23.49 ± 5.72 | 13.62 ± 2.52 | 43.59 ± 9.64 | 39.85 ± 8.73 |
| L-Tryptophan | 5.89 ± 0.77 | 6.87 ± 0.46 | 11.33 ± 2.42 | 15.44 ± 3.97 | 12.15 ± 4.99 | 10.22 ± 2.48 |
| D-Tryptophan | 0.36 ± 0.04 | 0.14 ± 0.03 | 0.45 ± 0.07 | 0.82 ± 0.11 | 0.34 ± 0.03 | 0.38 ± 0.11 |
| L-Cystine | 1.97 ± 0.31 | 1.32 ± 0.05 | 1.27 ± 0.15 | 1.84 ± 0.32 | 1.37 ± 0.21 | 1.58 ± 0.34 |
| D-Cystine | 0.55 ± 0.13 | 0.49 ± 0.09 | 0.62 ± 0.21 | 0.15 ± 0.02 | 0.14 ± 0.05 | 0.30 ± 0.08 |
| L-Lysine | 16.96 ± 2.78 | 10.37 ± 3.65 | 9.73 ± 2.46 | 15.44 ± 4.48 | 17.72 ± 5.46 | 20.01 ± 9.47 |
| D-Lysine | 0.07 ± 0.0 | 0.46 ± 0.07 | 0.10 ± 0.02 | 0.72 ± 0.07 | 0.41 ± 0.04 | 0.11 ± 0.03 |
| L-Tyrosine | 2.18 ± 0.12 | 1.15 ± 0.09 | 3.25 ± 0.15 | 2.09 ± 0.03 | 1.53 ± 0.25 | 1.74 ± 0.26 |
| D-Tyrosine | 0.05 ± 0.01 | 0.36 ± 0.07 | 0.07 ± 0.02 | 0.21 ± 0.04 | 0.32 ± 0.06 | 0.55 ± 0.15 |

**Figure S1**.- Comparison of detection sensitivity of amino acids between Q-TOF and Q-IM-TOF.
